# NETosis as a Pathogenic Factor for Heart Failure

**DOI:** 10.1155/2021/6687096

**Published:** 2021-02-23

**Authors:** Shuang Ling, Jin-Wen Xu

**Affiliations:** Institute of Interdisciplinary Medical Science, Shanghai University of Traditional Chinese Medicine, Shanghai 201203, China

## Abstract

Heart failure threatens the lives of patients and reduces their quality of life. Heart failure, especially heart failure with preserved ejection fraction, is closely related to systemic and local cardiac persistent chronic low-grade aseptic inflammation, microvascular damage characterized by endothelial dysfunction, oxidative stress, myocardial remodeling, and fibrosis. However, the initiation and development of persistent chronic low-grade aseptic inflammation is unexplored. Oxidative stress-mediated neutrophil extracellular traps (NETs) are the main immune defense mechanism against external bacterial infections. Furthermore, NETs play important roles in noninfectious diseases. After the onset of myocardial infarction, atrial fibrillation, or myocarditis, neutrophils infiltrate the damaged tissue and aggravate inflammation. In tissue injury, damage-related molecular patterns (DAMPs) may induce pattern recognition receptors (PRRs) to cause NETs, but whether NETs are directly involved in the pathogenesis and development of heart failure and the mechanism is still unclear. In this review, we analyzed the markers of heart failure and heart failure-related diseases and comorbidities, such as mitochondrial DNA, high mobility box group box 1, fibronectin extra domain A, and galectin-3, to explore their role in inducing NETs and to investigate the mechanism of PRRs, such as Toll-like receptors, receptor for advanced glycation end products, cGAS-STING, and C-X-C motif chemokine receptor 2, in activating NETosis. Furthermore, we discussed oxidative stress, especially the possibility that imbalance of thiol redox and MPO-derived HOCl promotes the production of 2-chlorofatty acid and induces NETosis, and analyzed the possibility of NETs triggering coronary microvascular thrombosis. In some heart diseases, the deletion or blocking of neutrophil-specific myeloperoxidase and peptidylarginine deiminase 4 has shown effectiveness. According to the results of current pharmacological studies, MPO and PAD4 inhibitors are effective at least for myocardial infarction, atherosclerosis, and certain autoimmune diseases, whose deterioration can lead to heart failure. This is essential for understanding NETosis as a therapeutic factor of heart failure and the related new pathophysiology and therapeutics of heart failure.

## 1. Introduction

Heart failure (HF) is a complex syndrome. Its typical symptoms are breathlessness, paroxysmal nocturnal dyspnea, reduced exercise tolerance, fatigue, tiredness, increased time to recover after exercise, and ankle swelling, resulting in decreased cardiac output and/or increased intracardiac pressure [1]. Currently, patients with HF are usually referred to as heart failure with reduced ejection fraction (HFrEF; LVEF < 40%), heart failure with midrange ejection fraction (HFmrEF; LVEF 40-49%), or heart failure with preserved ejection fraction (HFpEF; LVEF ≥ 50%) [1]. More than 64 million people in the world suffer from HF, with an estimated prevalence of 1-2% among adults in developed countries [2], while, in China, the HF prevalence of the Chinese adult population aged ≥35 years from 2012 to 2015 in China was 1.3% (estimated 13.7 million), which is a 44% increase compared to 2000. Among them, 1.4% of participants had left ventricular systolic dysfunction, and the prevalence of moderate/severe diastolic dysfunction was 2.7% [3]. The prevalence of HFpEF, HFmrEF, and HFrEF in China was 0.3%, 0.3%, and 0.7% [4]. Furthermore, among the 13687 patients with HF in 132 hospitals selected in the China-HF study from January 2012 to September 2015, the case fatality rate was 4.1% [4]. Moreover, the total number of HF patients in the world continues to increase because of the population growth and aging. HF has increased in low-income countries and shifted to HFpEF. Age, traditional risk factors for HF, sedentary lifestyle, and social deprivation are related to the occurrence of HF [5]. Many factors contributed to the development of HFpEF, such as inflammation, endothelial dysfunction, abnormal cardiac metabolism, cardiomyocyte hypertrophy, cardiac fibrosis, ventricular-vascular uncoupling, pulmonary hypertension, and chronotropic incompetence [6–13]. Although many studies have confirmed the correlation between inflammation and oxidative stress and the severity and prognosis of HF, except vitamin C, coenzyme Q10, and IL-1 antagonist anakinra, most of the clinical trials of anti-inflammatory and antioxidant therapy have been proved unsuccessful, indicating that we still have many unknowns about the mechanism of inflammation and oxidative stress in HF.

Neutrophils are powerful inducers of oxidative stress and inflammation in the immune system, but we know very little about their role and mechanism in HF. Recently, accumulating evidence shows that neutrophil extracellular traps (NETs) are an important way to be involved in the immune response. NETs are the last resort to control microbial infections released by neutrophils, and this unique cell death program of neutrophils is called “NETosis”. In this cell death process, citrullinated chromatin and bactericidal proteins from granules and cytoplasm are released and produce a network structure, which promotes the immobilization and killing of invading microorganisms in the extracellular environment. NETosis plays a vital role in host defense, autoimmunity, and blood coagulation [14, 15]. NETs can be activated through various disease-related stimuli, such as pathogens, antibodies and immune complexes, cytokines, microcrystals, and aging [16–19], and they also mediate tissue damage [20–22]. The induction of NETosis depends on the form of reactive oxygen species (ROS) via oxidative burst, and its main source is NADPH oxidase [23]. The structure of these NETs comprises various neutrophil-derived proteins such as myeloperoxidase (MPO), peptidylarginine deiminase 4 (PAD4), neutrophil elastase (NE), histones, neutrophil gelatinase-associated lipocalin (NGAL), proteinase-3, and DNA chains. In NETs, the enzymatic activity of MPO and NE may contribute to antibacterial activity or tissue damage [24, 25], and the MPO complex regulates NE release and actin dynamics [26]. Moreover, superoxide-dependent MPO-derived chlorinated lipids may be a mediator required for the formation of NETs (discussed in detail in the following section). PAD4 is important for NET-mediated antibacterial innate immunity [27], and it is also the nuclear button that triggers NETs in inflammatory diseases [28]. PAD4-mediated histone hypercitrullination promotes heterochromatin decondensation and chromatin unfolding to form NETs [29]. In addition to being a tool to defend against harmful foreign microbes, NETosis may also be the initiator of certain autoimmune and other noninfectious diseases [30]. NETs can also directly lead to acute and chronic inflammation, endothelial cell dysfunction, and thrombosis [31]. Some research results revealed that the formation of NETs occurs in the early or acute stage of noninfectious diseases, such as myocardial infarction and abdominal aortic aneurysms in cardiovascular diseases [32, 33], rheumatoid arthritis (RA) in autoimmune diseases, and antineutrophil cytoplasmic antibody- (ANCA-) associated vasculitis and inflammatory bowel disease [34–36]. Various facts indicate that NETosis may be involved in the process of HF, at least in the pathogenesis of heart failure-related diseases. Understanding, preventing, and targeting NETosis may contribute to the prevention and treatment of heart failure and improve the survival rate of patients.

## 2. NETosis Phenomena in HF

To our knowledge, the existence and function of NETs in HF, especially in HFpEF, have not been studied in detail. According to available data, in HF, especially in HFpEF, an aseptic inflammatory response with NETs as the core should be the necessary condition to enhance myocardial tissue damage, fibrosis, and ventricular remodeling.

More than a decade ago, several studies have reported that an increase in circulating MPO, as an independent risk factor, may play a key role in the occurrence and maintenance of chronic HF [37–39]. Furthermore, a recent study revealed that MPO is closely related to microvascular endothelial inflammation and dysfunction in HFpEF [40]. These findings indicate that circulating MPO derived from neutrophils may lead to the chlorination or nitration of protein tyrosine, causing protein dysfunction and vascular endothelial damage [39] and that the MPO-DNA complex may form NETs in HF tissues [41, 42]. NGAL, also known as lipocalin-2, is another component of NETs. As a siderophore-binding protein, it limits bacterial growth by sequestrating the iron-laden siderophore [43, 44]. Because it is also expressed in macrophages, endothelial cells, and cardiomyocytes, NGAL increase in the myocardial tissue of cardiac hypertrophy and experimental and clinical HF, although whether this source is related to NETs remains unclear [45–47]. Moreover, another index supports that neutrophils may be involved in the process of chronic HF. The neutrophil-to-lymphocyte ratio (NLR) is a risk factor for poor prognosis in elderly patients with chronic HF [48, 49] or HFpEF [50]. Furthermore, in animal models of HF, inflammatory infiltration of neutrophils was found in the myocardial tissue [51, 52]. These phenomena imply that NETs may promote the pathological process of HF.

A recent study reported that seipin/Bslc2 knockout mice, a model of Asian lean diabetes, developed HFpEF. The authors found that increased cardiac titin phosphorylation and reactive interstitial fibrosis associated with NETs lead to left ventricular stiffness. At the age of 23 weeks, cardiac hypertrophy and diastolic dysfunction were observed. The hearts of seipin/Bslc2 knockout mice displayed an increased end-diastolic pressure/volume relationship, increased end-diastolic pressure, and left atrial enlargement as well as exercise intolerance. NET was characterized by intact neutrophils with condensed nuclei (15 weeks old) and persisted as large amorphous extracellular structures, released DNA fibers decorated with MPO, and citrullinated histone (32 weeks old). By the age of 24 weeks, the mRNA and protein levels of the markers of cardiac inflammation and fibrosis, such as interleukin-6 (IL-6), tumor necrosis factor-alpha (TNF-*α*), intercellular adhesion molecule-1, collagen 1, alpha-smooth muscle actin, and transforming growth factor beta family, were significantly increased in the heart of seipin/Bslc2 knockout mice [53]. In heart tissue of seipin/Bslc2 knockout mice, hyperphosphorylation of JAK2 and downstream STAT3 was also found [53], suggesting that JAK2 signal can be connected to myocardial dysfunction and NETosis. In another JAK2^V617F^ allele conditional knock-in mouse model, clonal hematopoiesis accelerated pathological HF remodeling. After exposing these mice to stressors of coronary artery ligation-induced myocardial infarction and aortic constriction-induced pressure overload, researchers demonstrated that myeloid JAK2^V617F^ mice showed accelerated cardiac inflammation and remodeling, larger infarct size, and HF. In addition, on stimulation with LPS, THP-1 cells transduced with JAK2^V617F^ exhibited enhanced inflammatory responses, including transcripts of IL-6, IL-1*β*, TNF-*α*, and monocyte chemoattractant protein-1 (MCP-1), as well as AIM2 inflammasome component compared with JAK2^WT^ cells [54]. Another study using the same model showed that JAK2^V617F^ mutant neutrophils were prone to form NETs in vivo and lead to thrombosis events associated with myeloproliferative diseases. This study revealed that neutrophils expressing JAK2^V617F^ increased the protein expression of peptidylarginine deiminase 4 (PAD4) required for NETosis and that NETosis and thrombosis in vivo driven by JAK2^V617F^ require PAD4 [55]. Moreover, the PAD4 gene deletion in mice or the specific inhibitor GSK484 reduced myocardial infarction-induced neutrophil infiltration, citrulline histone H3 (citH3) expression, NET structure, and inflammatory cytokine secretion [56, 57]. A recent report reported that cardiac pressure overload induced NETosis and reduced left ventricular ejection fraction (LVEF) in WT mice but not in PAD4^−/−^ mice [58]. The deletion or activation of genes listed in Table 1 shows the phenotypes of mice with HF and NETosis. Therefore, NETosis may be involved in the development of HF and hence may be a new target for treatment.

## 3. NETosis in Diseases Inducing HF

**Figure 1:**
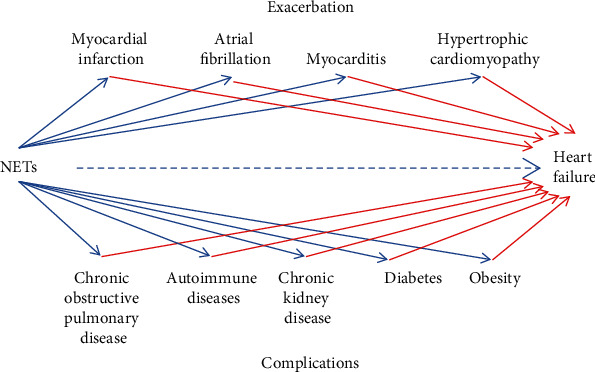
NETs' contribution to HF. NETs accelerate HF through promoting the deterioration of various heart diseases and systemic diseases. NETs: neutrophil extracellular traps.

Many diseases cause HF, such as myocardial infarction [59], atrial fibrillation [60], myocarditis [61], hypertrophic cardiomyopathy [62], chronic obstructive pulmonary disease [63], chronic kidney disease [64], diabetes [65], and autoimmune diseases. These examples show that many NET-related heart diseases and systemic diseases can together lead to HF (Figure 1). Here, the phenomenon and role of NETs in these diseases inducing HF will be discussed.

NETs may be linked to aseptic inflammation and microthrombosis. Studies have demonstrated that NETs and NET-mediated microthrombosis can be induced during myocardial ischemia-reperfusion (I/R), which lead to myocardial “no-reflow” and myocardial infarction [66]. Multiple clinical investigations have revealed that in patients with ST-segment elevation myocardial infarction (STEMI), double-stranded DNA (dsDNA), citH3, or NE increased in the culprit lesion site obtained through percutaneous coronary intervention compared with the femoral site [67, 68]. Cardiac magnetic resonance imaging performed for evaluating microvascular obstruction (MVO) revealed that with an increase in serum dsDNA, STEMI patients were likely to develop MVO and their acute infarction area was larger, suggesting that NET components are associated with infarcted size, ventricular function, and clinical outcomes in STEMI [69]. Both humoral and cellular components are involved in plaque formation and the development of atherosclerosis, a cause of myocardial infarction and HF. Regardless of lipid changes, studies have shown that inflammatory cytokines and cellular components contribute to myocardial infarction and cardiac death. The formation of NETosis is considered to be a very important way in aseptic inflammation [70] and an important participant in promoting thrombosis in complex plaques with intraplaque haemorrhages and in adjacent vascular tissues of atherosclerosis [71]. Moreover, patients with a high serum NLR had a significantly high cumulative probability of developing atrial fibrillation [72–74]. Experimental and clinical evidence revealed that neutrophil MPO is involved in atrial fibrillation pathogenesis. Under right atrial electrophysiological stimulation, MPO-deficient mice were protected from atrial fibrillation, which was reversed when MPO was restored; this finding indicated that MPO is a key prerequisite for myocardial remodeling, leading to increased susceptibility to atrial fibrillation [75]. Furthermore, NETosis provides new prognostic information for adverse cardiovascular events in patients with atrial fibrillation [76]. In addition, in a model of hypertrophic and hypertensive cardiomyopathy caused by pressure overload, neutrophils could be seen infiltrating into the heart of mice treated with transverse aortic constriction (TAC), exhibiting inflammation and cardiac dysfunction [51]. The enrichment analysis of its Gene Ontology terminology showed that common upregulated differentially expressed genes were mainly enriched in neutrophil chemotaxis and the extracellular fibril tissue [77].

Recently, many case reports have described the clinical observation and pathology of “fulminant myocarditis” of coronavirus disease 2019 (COVID-19). The patients had increased levels of troponin I, creatine kinase-MB, and brain natriuretic peptide. In addition to the high concentrations of IL-1*β*, IFN-*γ*, IFN-inducible protein-10, and MCP-1, patients with COVID-19 had high IL-6 levels. Echocardiography revealed that the heart was enlarged and the LVEF was significantly decreased. Bedside chest radiographs showed typical ground-glass changes indicative of viral pneumonia [78–83]. Although no direct data are available showing the presence of NETs in the heart, increased levels of cell-free DNA, MPO-DNA complex, and citH3 in the blood and involvement of NETs in lung autopsy indicate that COVID-19 activates NETosis [84–86]. Furthermore, individual plasma or serum of isolated COVID-19 patients triggers NETosis [84, 85]. COVID-19 exacerbates excessive tissue inflammation and thrombotic microangiopathy, thereby increasing mortality [86–88]. Coxsackievirus B3 (CVB3) is another major factor inducing viral myocarditis [89, 90]. In the acute phase of viral myocarditis, CVB3 internalization leads to increased secretion of IL-6, IL-1*β*, TNF-*α*, and IL-8/C-X-C motif chemokine ligand 8, and infected neutrophils released MPO and triggered NETosis in the presence of TNF-*α* [91]. Neutrophils recognized CVB3 mainly through endosomal Toll-like receptor- (TLR-) 8, and the infection triggered NF*κ*B activation [92]. Another report indicated that the mRNA expression of calcium-binding protein A8 and A9 (S100A8 and S100A9) derived from neutrophils in myocardial biopsy tissue of patients with CVB3-infected myocarditis increased by 13.0- and 5.1-fold, respectively. In CVB3-infected S100A8 and S100A9 knockout mice, left ventricular function improved and cardiac inflammatory and oxidative response decreased compared with those in wild-type CVB3-infected mice [93], suggesting that in addition to NETs, S100A8 and S100A9 derived from neutrophils play a key role in inducing myocarditis.

Patients with diabetes have an increased risk of HF, a high prevalence of HF and hospitalization, and poor prognosis [65]. Likewise, obesity and high-sensitivity cardiac troponin T (hs-cTnT) were both independently associated with incident HF, and individuals with severe obesity and a high hs-cTnT level had a significantly independent increased risk of incident HF [93]. This may be because increased cardiac output and hypertension lead to an increase in cardiac preload and afterload and left ventricular hypertrophy as well as the excessive deposition of myocardial collagen, abnormal protein glycosylation, and collagen cross-linking due to systemic inflammation. Therefore, obesity leads to HF through metabolic and inflammatory pathways [94]. Numerous studies have shown that the plasma or serum of patients with type 2 diabetes has increased levels of nucleosomes, human NE-DNA complex or MPO-DNA complex, IL-6, and TNF-*α* [95, 96]. Patients with previous myocardial infarction have increased citH3 and cell-free DNA levels [97]. Another study revealed that among 1572 patients with HF with decreased ejection fraction, 493 (31%) had diabetes. Compared with nondiabetic patients, diabetic patients have a high body mass index, severe symptoms and signs of HF, and hypertension history [98]. The authors performed the network analysis to determine whether the epidermal growth factor receptor and galectin-3 (GAL3) were the most prominent connecting proteins. The translation of these networks to biologic pathways revealed that diabetes was associated with inflammatory response and neutrophil degranulation [98]. By contrast, early studies have revealed that neutrophils infiltrate the capillaries of the subcutaneous and intra-abdominal fat tissue of obese patients [99, 100]. According to the plasma MPO-DNA complex, the NET level in the obesity group was higher than that in the healthy control group. In the medical history of patients with increased NETs, researchers observed increased thromboembolic events [101]. Prevention of NETosis with Cl-amidine, a cell-permeable pan PAD inhibitor, or dissolution of NETs with DNase restored endothelium-dependent vasodilation of the mesenteric arteries of diet-induced obese mice [102]. This indicates that obesity-induced NETs can impair the vasodilation function of mesenteric arterioles.

Accumulated evidence has confirmed that NETs are involved in the pathogenesis of autoimmune diseases such as RA, systemic lupus erythematosus (SLE), and vasculitis [103]. In clinical and biological observations, the overlap between NETs and RA, SLE, and vasculitis can usually be observed, which indicates that NETosis is the main triggering event of these autoimmune diseases. NETosis increases the possibility of association between citrullinated proteins or MPO autoantigens, and ANCA or anticitrullinated proteins/peptide antibodies (ACPA), triggering an autoimmune response [30, 104]. Numerous studies have revealed that RA and SLE can affect HF and other cardiovascular events (see review article References [105] and [106]). Furthermore, in RA patients, the plasma levels of ACPA and antimodified citrullinated vimentin antibody (AMCVA) are negatively correlated with LVEF [107]. Another study showed that higher ACPA and AMCVA levels are associated with higher adjusted mean left ventricular mass index (LVMI) compared with the lower antibody group [108]. An autopsy study of the heart found that the average and maximum intensity of anticitrulline staining in the myocardial interstitium of the RA group were 59% and 44% higher than those of the non-RA control group [109]. In a proteomics study targeting the citrullination of the heart protein of HF, 304 citrullination sites of 145 proteins were identified. Among them, citrullinated myosin decreased its intrinsic ATPase activity, and citrullinated tropomyosin leads to stronger F-actin binding; thus, the combined action of the two inhibits acto-heavy meromyosin ATPase activity, which indicates that citrullination of sarcomeric protein leads to an overwhelming reduction in Ca(2+) sensitivity in cardiomyocytes [110]. ANCA, as a specific antibody of proteinase-3 and MPO, is closely related to HF. In recent years, many cases have reported ANCA antibody-induced HFrEF, severe aortic regurgitation and mitral regurgitation, and a severe insufficiency of the aortic valve [111, 112]. One 20-year population-based cohort analysis recruited 58 patients diagnosed with ANCA-associated vasculitis (AAV); the risk of cardiovascular events (coronary artery disease, HF, and atrial fibrillation) was more than three times higher and the risk of cerebrovascular accident was eight times higher in AAV patients than in matched subjects [113]. The overlapping of antineutrophil citrullinated protein antibody or anti-MPO antibody with the heart damaged tissue suggests two possibilities: (1) with the infiltration of neutrophils in the cardiac tissue, NETosis is indeed a pathogenic factor of HF; (2) the existence of these two antibodies in the heart tissue is the embodiment of autoimmune disease eroding the heart function.

## 4. Promotion of NETosis and HF by Circulating Mitochondrial DNA

Mitochondrial DNA (mtDNA) leads to cardiac dysfunction in an aging heart. In elderly people aged >90 years, the plasma mtDNA level gradually increased with age, and the increase in the plasma mtDNA level was positively correlated with plasma TNF-*α*, IL-6, RANTES, and IL-1ra levels [114]. Moreover, the frequency of activated HLA-DR(+) neutrophils in elderly and frail elderly people was positively correlated with circulating mtDNA, which increased the expression of HLA-DR in neutrophils in a dose-dependent manner ex vivo [115]. Myocardial mtDNA content was positively correlated with the peripheral blood mtDNA content and left ventricular function in nonischemic HF patients [116]. Furthermore, during hypoxia reoxygenation, mtDNA induced inflammation and increased the expression of the proinflammatory cytokines IL-1*β* and TNF-*α* in mouse cardiomyocytes [117]. Circulating mtDNA may be used as a marker of increased mortality in patients with severe acute HF [118].

NETs represent extracellular structures that bind to and kill microorganisms. However, mtDNA released from damaged tissues can also induce NETosis, which may lead to tissue inflammation and thromboembolism. A decade-old study revealed that NETosis was induced by mtDNA released from cells [119]. mtDNA released from cancer, trauma, and tissue damage triggers NETosis through the TLR-9 signaling pathway [120, 121]. TLR-9, as one of the pattern recognition receptors (PRRs), senses unmethylated CpG dinucleotides that are relatively common in the genomes of most bacteria and DNA viruses but are suppressed and methylated in the genomes of vertebrate nuclei. The lysosomal localization of TLR-9 allows efficient detection of invading bacterial and viral nucleic acids while preventing accidental stimulation by CpG motifs within self-DNA [122]. Because mitochondria advance from prokaryotic bacteria, mtDNA retains molecular motifs similar to bacterial DNA. Therefore, TLR-9 can recognize mitochondrial unmethylated CpG sequences. The primary neutrophils of human and mouse express active and functional TLR-9 [123]. In the process of lung I/R, the release of acellular mtDNA triggers NETosis through the TLR-9 signal, and TLR-9 deficiency of neutrophils prevents mtDNA-induced NETosis [118, 119]. In HF, mtDNA escaping from autophagy cells can also induce myocarditis and dilated cardiomyopathy through TLR-9 signaling [124]. In a diastolic HF model established in cardiomyocyte-specific and inducible *SERCA2a* gene knockout C57Bl/6J mice, 4 weeks after conditional gene knockout, the mice were randomly given the TLR-9 agonist CpG-B. After 4 weeks, administering CpG-B shortened the life expectancy of *SERCA2a* gene knockout mice, reduced ventricular diastolic function, and increased heart and systemic inflammation, showing exacerbation of diastolic HF [125]. Stimulation of TLR-9 can induce inflammation and HF, whereas TLR-9 deficiency or long-term administration of the TLR-9 inhibitor E6446 or chloroquine can prevent left ventricular dilatation and cardiac insufficiency, fibrosis, and inflammation [126–128], providing a new perspective for the intervention and treatment of inflammatory-related diseases such as chronic HF. Another report revealed that cGAS-STING signaling is also included in NETosis induced by mtDNA. NETosis induced by mtDNA was attenuated in STING^−/−^ and TLR9^−/−^ mice in the area of tissue damage caused by I/R, suggesting that TLR-9 and STING pathways help mtDNA induce NET. Bone marrow neutrophils from STING^−/−^ and TLR-9^−/−^ mice showed a lower percentage of NET in mtDNA stimulation [123]. These findings provide a new perspective that circulating mtDNA may activate TLR-9- and cGAS-STING-mediated systemic and cardiac inflammation, chronic HF, and NETosis.

## 5. Damage-Associated Molecular Patterns and Cytokines Linking HF and NETosis

In addition to mtDNA, some other damage-associated molecular patterns (DAMPs) are used as important biomarkers and proinflammatory factors of HF, such as S100A8/A9 (calprotectin), high mobility box group box 1 (HMGB1), fibronectin extra domain A (FN-EDA), and GAL3 [129, 130]. Multiple studies have reported that serum S100A8/S100A9 levels derived from neutrophils were elevated in myocarditis [131], I/R injury [132], atrial fibrillation [133], or chronic HF [134], which can be used as a powerful potential biomarker. Thus, S100A8/A9 secreted by activated neutrophils is essential for proinflammatory function [135]. It is now understood that the induction of myocardial infarction leads to the rapid recruitment of neutrophils to the infarct, where they release the alarm protein S100A8/A9 and bind to TLR-4 to activate the naive neutrophil NLR family pyrin domain containing 3 inflammasome and promote IL-1*β* secretion. The released IL-1*β* stimulates the autonomous granulocyte production of hematopoietic stem cells in the bone marrow, which requires NETosis [136, 137].

In addition to the Kubota group, which used HMGB1 transgenic mice to show that cardiac nuclear HMGB1 exerts protective effects on myocardial infarction, cardiac hypertrophy, and HF [138–140], many studies have shown that HMGB1 is an independent predictor of death in HF [141–143]. By contrast, antagonistic HMGB1 box A, miR-129-5p, and the natural product HMGB1 inhibitor glycyrrhizic acid reduce infarct size and tissue damage of the heart; inhibit oxidative stress and inflammatory response; and prevent pressure overload-induced cardiac hypertrophy, heart fibrosis, and failure [144–147]. Importantly, HMGB1 promotes NETs and exacerbates tissue damage [148, 149]. Furthermore, soluble FN-EDA is a valuable biomarker in cardiac remodeling, and in patients with HF, the serum FN-EDA level was significantly elevated [150]. Studies have shown that in ApoE^−/−^ mice, FN-EDA promoted myocardial I/R injury, increased infarct size, elevated plasma cardiac troponin I levels, induced neutrophil infiltration and extracellular traps, and caused cardiomyocyte apoptosis [151]. GAL3 is another important component of DAMPs and a biomarker for cardiac fibrosis and failure. A high GAL3 level was associated with HF severity, such as a high New York Heart Association HF class; high systolic blood pressure; high creatinine, N-terminal prohormone of brain natriuretic peptide, IL-6, and C-reactive protein levels; and lower maximal oxygen consumption [152, 153], as well as increased left ventricular weight and echocardiographic changes in the left ventricular end-diastolic volume [154, 155]. The GAL3 level is a strong and independent predictor of unfavorable outcomes, which can predict the long-term mortality of patients with severe chronic HF [156, 157]. GAL3 has a higher predictive value in patients with preserved LVEF than in patients with reduced LVEF [153, 156, 157]. In contrast, some early studies have revealed that GAL3 facilitate neutrophil recruitment and has the ability to activate neutrophils [158–160]. Other two PRRs, TLR-4 and receptor for advanced glycation end products (RAGE), appear to be DAMP receptors. Neutrophils express TLR-4 and RAGE [161–164]. HMGB1, FN-EDA, and GAL3 activate the inflammation of neutrophils and microglial cells through TLR-4 activity [153, 165, 166]. Moreover, HMGB1 mediates subsequent tissue damage amplification through neutrophil recruitment induced by RAGE [167]. In myocarditis, HMGB1 and its major receptor RAGE seem to be key factors in the pathogenesis of TnI-induced experimental autoimmune myocarditis (EAM) [168].

**Figure 2:**
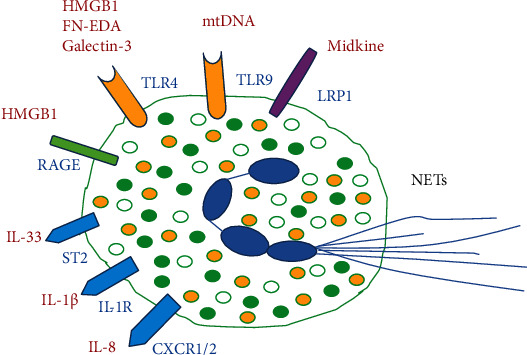
Initiation of NETs by PRRs. Various DMAPs and cytokines activate NET formation through PRRs. FN-EDA: fibronectin extra domain A; HMGB1: high mobility box group box 1; LRP1: LDL receptor-related protein 1; mtDNA: mitochondrial DNA; PRRs: pattern recognition receptors; RAGE: receptor for advanced glycation end products; ST2: a receptor for IL-33; TLR: Toll-like receptor.

Dilated cardiomyopathy caused by myocarditis can develop into HF. A recent study reported that midkine induced cardiac inflammation by promoting neutrophil trafficking and NETosis in myocarditis. In EAM in mice, midkine as a target can not only inhibit NETosis and neutrophil infiltration in vivo but also reduce myocardial fibrosis and maintain cardiac function [169]. Midkine is a proproliferation and proinflammatory cytokine. A clinical investigation indicated that the serum midkine concentration of HF patients was significantly higher than that of controls. Patients with cardiac events had a higher midkine concentration than did patients without cardiac events [170]. After TAC, the midkine expression level was increased in the kidney and lungs but not in the heart. After TAC, compared with wild-type mice, transgenic mice with cardiac-specific overexpression of midkine showed more severe cardiac hypertrophy and dysfunction and a lower survival rate [171]. Moreover, renal ischemic injury could increase midkine expression in the proximal tubules of the kidney, leading to the recruitment of neutrophils to the tubule interstitium. The knockout of the midkine gene inhibited neutrophil infiltration [172] and reduced myocardial hypertrophy induced by subtotal nephrectomy compared with wild-type mice [173]. Many studies have demonstrated that LDL receptor-related protein 1 (LRP1) is an endocytic receptor of midkine [174, 175], and it facilitates neutrophil adhesion and trafficking through the interaction between LRP1 and *β*2 integrins during acute inflammation [176–178]. More importantly, midkine initiates NETosis through interaction with LRP1 [169]. The neutrophil activity mediated by DAMPs is shown in Table 2 and Figure 2. Furthermore, in a relatively early period, many studies have confirmed that high sensitivity C-reactive protein (hs-CRP) can be used as a potential indicator of risk stratification in HF patients [179]. The plasma hs-CRP level gradually increases with the deterioration of LV diastolic dysfunction [180]. Compared with HFrEF patients, CRP predicts mortality in HFpEF patients is significantly enhanced [181]. Recent data indicate that the higher the plasma hs-CRP is, the higher the mortality of HF patients is [182]. Thirty years ago, it was discovered that hs-CRP can bind to neutrophils [183]. Then, it was confirmed that CRP binds to IgG Fc*γ*RI (CD64) and IgG Fc*γ*RII (CD32) high-affinity receptors [184, 185]. Recent research results show that compared with patients with low CRP with STEMI, patients with high CRP are accompanied by elevated circulating IL-1*β*, NETosis, and NET-associated tissue factor plasma levels [186]. The serum CRP level of patients with HF or HF+type 2 diabetes (T2DM) is significantly higher than that of the healthy control group, and the NET release rate from HF or HF+T2DM patients is faster than that of T2DM and healthy control groups without stimuli [187]. Earlier studies pointed out that CRP stimulates the formation of oxidative bursts as a necessary condition for NET release [188–190]. Besides, studies from a decade ago have found a significantly higher level of platelet-derived soluble CD40L in patients with chronic HF [191, 192]. Recent researches also discovered a correlation between soluble CD40L and the release of NETs and the increase of oxidative burst [193]. The above summarized references indicate that DAMPs, soluble CD40L derived from platelets, and CRP in the adjacent tissues activate the formation of NETs.

## 6. Role of Oxidative Stress in HF and NETosis

**Figure 3:**
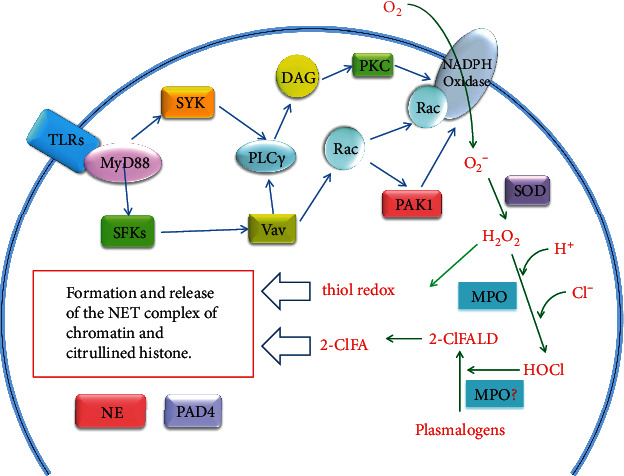
Possible pathways for the formation of NETs activated by oxidative stress. 2-ClFA: 2-chlorofatty acid; 2-ClFALD: 2-chlorofatty aldehyde; DAG: diacylglycerol; MPO: myeloperoxidase; NE: neutrophil elastase; PAD4: peptidylarginine deiminase 4; PAK1: P21 (Rac)-activated kinase 1; PLC*γ*2: a calcium-dependent phospholipase; PKC: protein kinase C; Rac: Rac GTPases, a small G-protein; SFKs: Src family protein tyrosine kinases, which include eight members: c-Src, c-Yes, Fyn, c-Fgr, Lyn, Hck, Lck, and Blk; SOD: superoxide dismutase; SYK: spleen tyrosine kinase; TLRs: Toll-like receptors; Vav: a guanine nucleotide exchange factor.

As we all know, oxidative stress is closely related to inflammation. A large amount of evidence has shown that inflammation plays an important role in the onset and development of cardiovascular diseases [194]. Similarly, numerous evidences also fully reveal that oxidative stress and ROS-related low-grade chronic inflammation affect the occurrence and development of cardiac systolic and diastolic function, cardiac hypertrophy, and remodeling [195–197]. NETosis is a form of inflammation and cell death, while the formation of NET requires an oxidative burst of neutrophils [198, 199]. NETosis caused by various stimuli depends on the production of the oxidative burst, which is mainly produced by NADPH oxidase activation [200], but some studies have found that mitochondrial oxidative stress is indispensable for this process [201–203]. Previous studies have confirmed that the oxidative burst in the generation of NETosis involves various signals via NADPH oxidase, such as Rac and Pak signals [204], protein kinase C [205], Src family kinases [206], spleen tyrosine kinase [207], and Vav-PLC*γ* [208] (Figure 3). Oxidative burst is mediated by a variety of PRRs, such as TLR2, TLR3, TLR4, and TLR 9 [209–211]. Moreover, RAGE also activates oxidative burst in diabetes [212]. Interestingly, the priming of the neutrophil oxidative burst requires IL-8 to induce sequential recruitment of NADPH oxidase components into lipid rafts [213]. In this process, phospholipase D is also activated by IL-8 [214].

Recently, a study reported that the redox imbalance of neutrophils during the oxidative burst promotes the generation of NETosis. The authors believed that the generation of NETosis is partly regulated by the changes in the cytosolic thiol redox homeostasis in neutrophils and depends on the circumstances under which NETosis is generated [215]. Meanwhile, more research focuses on the role of MPO and MPO-derived HOCl in the uncontrolled NET formation (NETosis). Researchers found that completely neutralizing extracellular ROS is not enough to block PMA-triggered NETosis generation, but removing MPO-related ROS in azurophilic particles effectively prevents the suicidal NETosis [216]. Furthermore, in MPO-deficient neutrophils, the extracellular addition of MPO still cannot rescue the formation of NETosis, indicating that the intracellular granules are involved in [216]. MPO is the peroxidase in the lysosomal cyanophilic granules of neutrophils, and the target of HOCl derived from MPO has been a topic of concern in recent years. Up to now, it is known that plasmalogen phospholipids can be oxidized by HOCl to 2-chlorofatty aldehyde (2-ClFALD). Up to now, it is known that 2-ClFALD has four metabolic pathways, which can be oxidized to 2-chlorofatty acid (2-ClFA), reduced to 2-chlorofatty alcohol, formed Schiff base adducts with proteins and amines, or reacted with glutathione through nucleophilic attack of *α*-chlorinated carbon [217]. Because the plasma membrane of neutrophils, endothelial cells, vascular smooth muscle cells, myocardial cells and nerve cells enriched with plasmalogens as the source of 2-ClFALD, this provides the possibility of plasmalogen reacting with HOCl in biological systems [218]. In the heart of myocardial infarction rats, *α*-chloro fatty aldehyde 2-chlorohexadecanal (2-ClHDA), a 16-carbon chain chlorinated fatty aldehyde, accumulated in the hearts of rats with left anterior descending artery occlusion. Compared with sham-operated rats, levels of 2-ClHDA and neutrophil infiltration in myocardium of surgical infarction rats were increased [219]. In recent years, studies have revealed that human neutrophils are treated with physiological levels of 2-ClFAs to form NET structure, which is characterized by the binding of MPO to DNA and NE redistribution to the area around the nucleus. 2-ClFA can also induce NETosis of bone marrow-derived neutrophils in MPO-deficient mice [220] (Figure 3). Many studies have explored the effects of 2-ClFALD and 2-ClFA on the function of neutrophils, vascular endothelial cells, monocytes, and blood vessels, suggesting that MPO-derived chlorinated lipids can cause inflammation and vascular tension changes, and contribute to the occurrence and development of cardiovascular diseases [217].

Serum albumin is a powerful predictor of death or hospitalization in patients with HFpEF. Low serum albumin is the link between higher myocardial extracellular volume and higher levels of N-terminal pro-B-type natriuretic peptide (NT-proBNP) [221]. Low serum albumin is also associated with arterial stiffness, diastolic dysfunction, pulmonary hypertension, and inflammatory biomarkers such as white blood cell count, IL-6, and TNF-*α* in patients with HFpEF [222]. In patients with diabetic nephropathy, hypoalbuminemia, systemic inflammation, and oxidative stress are common. However, due to oxidized albumin, hypoalbuminemia in these patients may be underestimated, and increased oxidized albumin may lead to accelerated cycle between oxidative stress and neutrophil activation [223]. Recently, it has been reported that the oxidation of albumin, a major source of free thiol, is sufficient to trigger NETosis through accumulation of reactive oxygen species within neutrophils in pulmonary cancer metastasis, because the oxidation of albumin-derived free thiol leads to the redox imbalance in the blood [224]. The free thiol derived from albumin reflects the systemic redox state in the circulation. Studies have shown that patients with chronic HF with higher mean serum-free thiol concentration are younger, have better kidney function, have lower levels of NT-proBNP, and have reduced rehospitalization rates and increased patient survival rates [225]. These studies suggest that the redox imbalance of albumin oxidation through the serum-free thiol reaction is a way to trigger NETosis.

## 7. Role of SIRT3 in HF and NETosis

SIRT3 is a major mitochondrial deacetylase and an important factor in maintaining cardiac mitochondrial bioenergy. Multiple studies have revealed that SIRT3 deficiency or decreased expression can induce the excessive acetylation of pyruvate dehydrogenase, ATP synthase, cyclophilin D, and mitochondrial dynamic like GTPase OPA1; increase the opening of mitochondrial permeability transition pore; and change the process of oxidative phosphorylation, resulting in mitochondrial dysfunction [226–228]. Furthermore, SIRT3 deficiency or decreased expression caused changes in cardiomyocyte glycolysis, myocardial hypertrophy, and fibrosis and low cardiac reserve capacity, which impaired diastolic function and induced senile HF [226–228]. SirT3 expression was lower in STEMI and obese patients and hypertension model animals than in healthy donors [171, 227, 229]. In the LPS-excited laser-induced carotid artery thrombosis model, compared with the Sirt3^+/+^ wild-type control group, the thrombotic occlusion time in Sirt3^−/−^ mice was reduced by half [171]. Moreover, *ex vivo* LPS-induced NETosis was increased in Sirt3^−/−^ bone marrow-derived neutrophils, indicating that SIRT3 deletion in the inflammatory environment affected NETosis and arterial thrombosis in mice [171]. Another report indicated that long-term exposure to indoxyl sulfate, a uremic toxin that induces HF, promoted arterial thrombosis by reducing levels of SIRT1 and SIRT3 in the aorta [230].

## 8. Effect of NETosis on HF via Thromboembolism

In recent years, several studies have shown that NET promotes thrombus formation [55, 231, 232]. Neutrophil-derived PDP4 promotes vWF-platelet string formation [233]. The dense chromatin released by neutrophils constructs an extracellular DNA network. This network forms scaffolds in inflammatory vessels; promotes platelet adhesion, activation, and aggregation; absorbs red blood cells; leads to fibrin deposition; and induces thrombosis [234]. During NETosis, the released DNA also forms a network structure and interacts with vWF to promote the adhesion and activation of platelets [234]. Studies have shown that the new blood nucleic acid scavenger can inhibit thrombosis without increasing bleeding, which reversely demonstrated the role of the neutrophil DNA network structure in thrombosis [235]. Furthermore, neutrophils recruited to the tissue damage area release the specific alarm protein S100A8/A9 [236, 237] and stimulate their naive neutrophils to mature in a cell-autonomous manner [238]. Moreover, S100A8/A9 regulates thrombosis by binding to CD36 on platelets [239].

**Figure 4:**
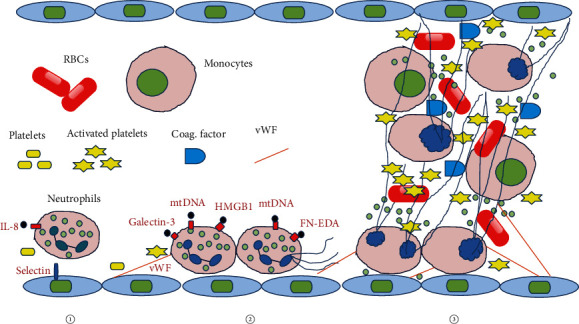
Possible mechanism of DMAPs activating NET-dependent thrombosis. (1) Migration of neutrophils driven by the chemokine receptor CXCR2 and ligand IL-8. (2) Various DMAPs induce thrombosis by activating NETs. (3) With the participation of activated platelets, endothelial cells, monocytes, red blood cells, and blood coagulation factors, neutrophils promote thrombosis. Coag. Factor: coagglutination factor; FN-EDA: fibronectin extra domain A; HMGB1: high mobility box group box 1; mtDNA: mitochondrial DNA; RBCs: red blood cells; vWF: von Willebrand factor.

Recent studies have revealed that DMAPs that induce NETs also promote thrombosis, such as HMGB1, FN-EDA, GAL3, and C-X-C motif chemokine receptor 2 (CXCR2). In addition to activating neutrophils, HMGB1 also regulates platelet activation, granule secretion, adhesion, and diffusion through the TLR-4 pathway on platelets [240]. Similar to HMGB1, FN-EDA^−/−^ mice showed prolonged time for thrombosis and complete occlusion, and the thrombus growth rate was significantly reduced compared with FN-EDA^+/+^ mice. TLR-4 gene deletion reversed accelerated thrombosis in FN-EDA^+/+^ mice [241]. Although no direct evidence exists regarding arterial thrombosis, in the deep venous thrombosis (DVT) model of GAL3^+/+^ mice, galectin-3-binding protein (GAL3BP) and GAL3 were localized in the vein wall, red blood cells, and platelets, whereas only GAL3 was expressed in neutrophils. GAL3 increased significantly during early DVT, and GAL3BP/GAL3 was co-located at the interface between neutrophils and endothelial cells. Some neutrophils attached to the vein wall, whereas the number of activated neutrophils in the vein wall of GAL3^−/−^ mice significantly decreased. Thrombus size was associated with increased levels of GAL3 and IL-6 in the venous wall [242]. IL-8, neutrophil-activating peptide-2, and growth-related protein alpha trigger cell activation through binding to specific and different amino acid residues of CXCR2, which includes the induction of neutrophil migration [243]. Neutrophils migrate through a CXCR2-dependent mechanism to accumulate in the thrombus and stimulate the release of NETs to increase the frequency and size of thrombus [244]. The possible mechanism of DMAPs activating NET-dependent thrombosis is shown in Figure 4. As early as 1981, aortic thrombosis was reported to be present in congestive HF [245], and then, it was believed to be related to vWF and platelet receptor protein of glycoprotein [246]. In clinical practice, HF is considered to be associated with an increased incidence of thromboembolic events [247], and many causes of HF death are also related to thromboembolic events [248].

## 9. Cardioprotective Effect of NETosis-Related Inhibitors

Neutrophil specific enzymes, MPO, PAD4, and NE, play a key role in the formation of NETs. Up to now, PAD4 inhibitors such as BMS-P5, GSK484, GSK199, and cl-amidine and MPO inhibitors such as PF-1355, PF-06282999, INV-315, and AZD5904 have been developed. MPO is a peroxidase containing heme, mainly expressed in neutrophils and a small amount in monocytes, where it catalyzes the production of reactive oxygen intermediates such as hypochlorous acid (HOCl) as antibacterial armory from hydrogen peroxide and Cl^−^ ions. However, MPO is also associated with oxidative stress and tissue damage resulting from the potent oxidant HOCl, which plays a key pathogenic event in a variety of inflammatory states, including cardiovascular disease. MPO inhibitors, such as KYC, seem to prevent PMA-stimulated neutrophil HOCl without affecting superoxide production [249]. Another way to eliminate NET formation is PAD4 inhibitor. A report claimed that the PAD4 inhibitor Cl-amidine can inhibit leukocyte activation, oxidative burst from overactive leukocytes, and subsequent DNA damage of target epithelial cells in vitro and in vivo in an ulcerative colitis mouse model [250]. PAD4 and the cytoplasmic subunits of NADPH oxidase, p47phox and p67phox, can form a physical association, leading to citrullination of the cytoplasmic subunits of NADPH oxidase and oxidative explosion. The small molecule PAD4 inhibitors, such as BB-Cl-amidine and GSK484, can destroy the complex of PAD4 and the cytoplasmic subunits of NADPH oxidase and reduce the oxidative burst [251].

Recently, several studies have reported the effect of PAD4 inhibitors on cardiovascular disease (Figure 2). In a mouse myocardial ischemia model, GSK484, a PAD4 inhibitor, reduces infarct size and cardiomyocyte apoptosis, promotes the improvement of cardiac function, ameliorates cardiac neutrophil infiltration and NET formation, depresses PAD4 expression and cit-H3 level, and inhibits secretion of inflammatory cytokines [57]. Another study also supports that Cl-amidine abrogates NET formation, decreases arterial thrombosis, and limits injury in a mouse model of myocardial infarction [252]. Similar results have been observed in the ApoE^−/−^ atherosclerosis mouse model. Treatment with Cl-amidine for 11 weeks decreases the atherosclerotic lesion area, reduces the recruitment of neutrophils and macrophages to the artery and prevents the formation of NET, and downregulates interferon-*α* expression in the arteries. In addition, Cl-amidine treatment delays carotid artery thrombosis in a photochemical injury model [253]. However, although the MPO inhibitor PF-06282999 cannot change the macrophage content and white blood cell homing in atherosclerotic plaques, it can reduce the necrotic core area, suggesting that PF-06282999 promotes the stability of atherosclerotic lesions in the LDLR^−/−^ mouse model of atherosclerosis [254]. Likewise, another MPO inhibitor INV-315 results in reduced plaque burden and enhanced cholesterol efflux, decreased aortic iNOS gene expression, superoxide production, and nitrotyrosine content, and improved endothelial function of acetylcholine [255]. Further, gavage administration of the MPO inhibitor PF-1355 for 7 days also inhibits the increase in MPO activity in the myocardial infarction area of mice, reduces the number of inflammatory cells, and slows down the expansion of the left ventricle. After 21 days of administration, the mouse heart function and remodeling were improved (Figure 2) [256]. Moreover, specific NE from neutrophils is another key enzyme involved in the formation of NETs. Research reports have shown that its inhibitors Sivelestat and SSR69071 can improve myocardial injury caused by endotoxin or/and ischemia-reperfusion (Figure 2), respectively [257, 258]. Sivelestat treatment effectively maintained the structure of mouse vascular endothelium and endothelial glycocalyx [257], while SSR69071 can reduce infarct size [258]. Additionally, PAD4 inhibitor Cl-amidine or BB-Cl-amidine shows a good therapeutic effect on heart failure-associated autoimmune diseases such as rheumatoid arthritis, lupus erythematosus, MPO-ANCA-associated vasculitis, and Behçet's disease [259–263]. The MPO inhibitor PF-1355 also suppresses immune complex vasculitis and glomerular basement membrane nephritis [264].

Statins and metformin may effectively improve heart failure clinically (see review article References [265–268]). In recent years, a large number of studies have reported that both statins and metformin have shown effective anti-inflammatory effects. Evidence shows that simvastatin and metformin can inhibit the formation of NETs [269, 270]. Clinically, rosuvastatin significantly attenuates the plasma MPO level, platelets, and circulating neutrophils and monocytes [271, 272]. Similarly, metformin also decreased plasma MPO levels, reduces the number of peripheral blood neutrophils, and prevents infiltration to myocardium in clinical and animal experiments [273, 274]. These pharmacological and clinical studies have shown that resisting the formation of NETs is beneficial for improving heart failure.

## 10. Conclusion

In short, DAMPs, such as mtDNA, HMGB1, FN-EDA, and GAL3, released by damaged organs may activate neutrophils and form NETs through TLR-4/9 and RAGE, which requires the participation of oxidative stress, thereby causing or aggravating cardiac inflammation and fibrosis. NETs promote thrombosis of coronary microvessels and affect cardiac function. In some studies, neutrophil-specific MPO and PAD4 ablation or inhibition could improve the disease progression of HF, myocardial infarction, and atrial fibrillation, which all illustrate the fact that NETosis is a pathogenic factor of HF. Recently, Abrams et al. [275] have developed a new assay that may independently predict diffuse intravascular coagulation and mortality in critically ill patients caused by neutrophil extracellular traps. These facts are important for understanding the new pathophysiology of HF and will contribute toward the development of therapeutics and pharmacy.

## Figures and Tables

**Table 1:** Effect of gene deletion or activation on NETosis and HF.

Gene	NETosis	Heart failure
Seipin/Bslc2	Compared with 15-week-old SKO (seipin/Bscl2 deficiency in developing adipocytes) mice, 32-week-old SKO mice had hearts with large amorphous NET structures, which released MPO and citrullinated histone-modified DNA fiber [53].	Lipodystrophic mice with seipin/Bscl2 deletions were lean and diabetic and showed the main clinical manifestations and characteristics of HFpEF. Increased myocardial titin phosphorylation and reactive interstitial fibrosis associated with NETs led to left ventricular stiffness and HF [53].
JAK2	JAK2^V617F^-driven myeloproliferative neoplasm mouse models had a NET-rich, prothrombotic phenotype, whereas the JAK inhibitor ruxolitinib and DNase reduced the rate of venous thrombosis induced by the mouse model [55].	At the 14th day of the myocardial infarction experiment, mice expressing JAK2^V617F^ had enlarged infarct size, increased fibrosis, and significantly increased IL-6 and IL-1*β* levels. Ly6Chi monocytes, neutrophils, and macrophages in the infarcted area also showed an increasing trend. In another HF experiment in mice, 8 weeks after the operation, the heart mass and lung weight of the JAK2^V617F^ experimental group increased significantly compared with the JAK2^WT^ experimental group. Echocardiography revealed that the JAK2^V617F^ experimental group displayed significantly increased cardiac posterior wall thickness and a progressive reduction of fractional shortening. TAC treatment induced more severe myocardial hypertrophy and cardiac fibrosis in the JAK2^V617F^ group than in the JAK2^WT^ group [54].
PAD4	After receiving ascending aorta contraction, wild-type mice showed neutrophils in the early stage of NETosis, extracellular H3Cit derived from Ly6G^+^ neutrophils, and citrullination of histone H3 in the nucleus; however, PAD4^−/−^ mice did not show these characteristics [58].	The heart functions of PAD4^−/−^ mice in the early stage (3 d and 7 d) and late stage (28 d) were protected from the LVEF decrease observed in WT mice. Compared with WT animals, the collagen content of PAD4^−/−^ mice was low. Furthermore, PAD4^−/−^ mice had lower perivascular fibrosis than did WT mice [58].
MPO	PMA induced significant ecDNA release in WT neutrophils but not in MPO^−/−^ neutrophils compared with controls. However, 2-chlorofatty acids, as lipid metabolites and mediators of MPO, can bypass its physiological formation, resulting in a significant release of ecDNA from WT and MPO^−/−^ neutrophils [220].	Compared with wild-type mice, the peri-infarct zone of the ventricle of MPO-deficient (MPO^−/−^) mice showed less obvious conductivity heterogeneity and deceleration. Furthermore, MPO^−/−^ mice showed decreased ventricular postischemic fibrosis, reflecting reduced accumulation of myofibroblasts [276].
CXCR2	Neutrophils cocultured with tumor cells and stimulated with CXCR2 receptor agonists, such as IL-8 and induced NETs [277].	CXCR2 deficiency blocked angiotensin II-induced cardiac hypertrophy, fibrosis, and inflammation [278].
MDK	The targeted midkine (MDK) not only inhibited the NETosis and infiltration of neutrophils in vivo but also reduced fibrosis occurrence in EAM and preserved the contractile function of the heart [169].	The cardiac-specific MDK-overexpressing mice (MDK-TG) showed more severe cardiac hypertrophy and dysfunction and a lower survival rate after TAC than did WT mice [171].
Sirt3	Ex vivo LPS-induced NETosis increased in Sirt3^−/−^ bone marrow-derived neutrophils. In vivo time to thrombotic occlusion in Sirt3^−/−^ mice was reduced by half compared with Sirt3^+/+^ wild-type controls [226].	Compared with wild-type mice, Sirt3^−/−^ mice had a short lifespan. With increasing age, Sirt3^−/−^ mice showed cardiac hypertrophy, fibrosis, and cardiac insufficiency. Sirt3 deficiency changed myocardial mitochondrial bioenergy, leading to the acetylation of OPA1, the target of Sirt3. Transfection of the deacetylated Opa1 gene improved the heart reserve capacity and protected the heart from hypertrophy and fibrosis [279].
RIPK3	Ripk3^−/−^ neutrophils lacked Sytox green nucleic acid stain uptake into the nucleus, chromatin decondensation, NET formation, PicoGreen^+^ DNA release, and plasma membrane rupture as compared with Ripk3^+/+^ neutrophils [280].	The haploinsufficiency of RIPK3 significantly attenuated Cops8-specific knockout mice in cardiomyocyte- (Cops8CKO-) induced cardiomyocyte necrosis and delayed mouse premature death [281].

**Table 2:** Effect of inhibitors of enzymes activating NETosis on heart failure-related diseases.

Classification	Therapeutic or experimental purposes	Drug	Animal	Model	Administration	Dose	Experimental period	Effects	References
PAD4 inhibitor	Myocardial infarction	GSK484	20–25 g male C57BL/6 mice	Permanent ligation of the left anterior descending coronary artery was performed to induce myocardial infarction in mice	Intraperitoneal injection (3 days before MI and 2 days after MI)	4 mg/kg per day	3 days before MI and 2 days after M I	Using GSK484 can moderately preserve the tissue structure and myocardial integrity of the ventricle after MI, reduce the infarct size, and reduce the level of myocardial CK-MB, LDH, and cTnT in the serum. PAD4 inhibition also effectively protects cardiomyocytes from MI-induced NET formation and secretion of inflammatory cytokines IL-1*β*, IL-6, and TNF-*α* and reduces cardiomyocyte apoptosis caused, thereby improving the overall heart function	[57]
	Myocardial infarction and thrombus formation	Cl-amidine	C57BL6/J mice aged 8-14 weeks and weighing 21-25 g	(1) Use of wire injury or topical application of FeCl_3_ to induce thrombus in the mouse carotid artery; (2) the LAD was ligated and the PE-10 tube was withdrawn after 60 minutes to create a mouse cardiac ischemia-reperfusion model	In the myocardial ischemia-reperfusion model, Cl-amidine was administered intraperitoneally at the onset of ischemia and at time of reperfusion. A third dose of Cl-amidine was administered 12 hours after reperfusion	10 mg/kg		FeCl_3_-induced mouse arterial thrombosis is highly consistent with the immune cell composition of coronary artery thrombosis in patients with myocardial infarction. Neutrophils are the most abundant cell type, and there are NETs and coagulation factors. Cl-amidine abrogated NET formation, reduced arterial thrombosis, and limited injury in a model of myocardial infarction	[252]
	Atherosclerosis	Cl-amidine	Apolipoprotein-E (Apoe)^−/−^ mice	Feeding high-fat food (42% from fat)	Daily subcutaneous injection	10 mg/kg per day	Beginning at 7 weeks and through euthanasia at 18 weeks	Pharmacological inhibition can prevent the formation of NET, reduce the area of atherosclerotic lesions, and delay the time of carotid artery thrombosis, accompanied by decreased recruitment of networked neutrophils and macrophages to the artery, and decreased expression of interferon-*α*	[253]
MPO inhibitor	Atherosclerosis	PF-06282999	LDLR^−/−^ male mice	Feeding a western diet (0.2% cholesterol), *ad libitum*	Daily gavage	5 or 15 mg/kg	Starting from 8-10 weeks for 7-16 weeks	After 4 weeks of treatment with MPO inhibitors, it reduces the necrotic core area of the aortic root and decreases the activity of MPO and the uptake of [^18^F]-fluoro-deoxy-glucose in the aorta, as an indicator of plaque load and inflammation, but the inhibitory effect of MPO will not change the homing of white blood cells	[254]
	Myocardial infarction	PF-1355	8-12 weeks old female C57Bl/6J mice	For ischemia-reperfusion injury (IRI) model, left coronary artery was transiently ligated for 30 minutes and then the ligation suture was removed	Twice daily by oral gavage	50 mg/kg	21 day	MPO inhibition for 7 days resulted in decreased MPO and CD11b expression, and inflammatory Ly6C^high^ monocytes. Continuous MPO inhibition for more than 21 days can significantly improve the ejection fraction, the end-diastolic volume/end-systolic volume ratio, and the effect of cardiac magnetic resonance on the quality of the left ventricle	[255]
NE inhibitor	Endotoxin-induced myocardial injury	Sivelestat	9-12-week-old male mice	20 mg/kg LPS intraperitoneal injection to induce endotoxin	Intraperitoneal injection	0.2 mg/kg	48 h after LPS treatment	The survival rate of mice injected with sivelestat was significantly higher, while the levels of serum troponin I and IL-6 were significantly lower than those of the control group. The vascular endothelium of mice treated with sivelestat clearly showed that the endothelial glycocalyx was well preserved at the ultrastructural level	[256]
	Ischemia-reperfusion injury	SSR69071	Male New Zealand white rabbit, weighing 2-3 kg	Coronary artery occlusion for 30 min followed by reperfusion for 120 min	Intravenous injection 15 min before coronary ligation or 25 min after coronary ligation (5 min before reperfusion)	1, 3, and 10 mg/kg		Elastase activity in the heart was significantly increased in ischemia-reperfusion animals. The activity of elastase was significantly decreased after treatment with ssr69071 before reperfusion. Ssr69071 treatment significantly reduced myocardial infarction size	[257]

LAD: left anterior descending artery; LPS: lipopolysaccharides; MI: myocardial infarction; MPO: myeloperoxidase; NE: neutrophil elastase; PAD4: peptidyl arginine deiminase-4; PCI: percutaneous coronary intervention.

## Abbreviations

2-ClFA: 2-Chlorofatty acid
2-ClFALD: 2-Chlorofatty aldehyde
2-ClHDA: Alpha-chloro fatty aldehyde 2-chlorohexadecanal
ACPA: Anticitrullinated proteins/peptide antibody
AMCVA: Antimodified citrullinated vimentin antibody
ANCA: Antineutrophil cytoplasmic antibody
citH3: Citrulline histone H3
COVID-19: Coronavirus disease 2019
hs-CRP: High-sensitivity C-reactive protein
hs-cTnT: High-sensitivity cardiac troponin T
CVB3: Coxsackievirus B3
CXCR2: C-X-C motif chemokine receptor 2
DAMPs: Damage-associated molecular patterns
dsDNA: Double-stranded DNA
DVT: Deep venous thrombosis
EAM: Experimental autoimmune myocarditis
FN-EDA: Fibronectin extra domain A
GAL3: Galectin-3
GAL3BP: Galectin-3-binding protein
HF: Heart failure
HFpEF: Heart failure with preserved ejection fraction
HMGB1: High mobility box group box 1
IL-1*β*: Interleukin-1beta
IL-6: Interleukin-6
IL-8: Interleukin-8
I/R: Ischemia-reperfusion
LRP1: LDL receptor-related protein 1
LVEF: Left ventricular ejection fraction
LVMI: Left ventricular mass index
MCP-1: Monocyte chemoattractant protein-1
MPO: Myeloperoxidase
mtDNA: Mitochondrial DNA
MVO: Microvascular obstruction
NE: Neutrophil elastase
NETs: Neutrophil extracellular traps
NETosis: Neutrophil extracellular trap formation
NGAL: Neutrophil gelatinase-associated lipocalin
NLR: Neutrophil-to-lymphocyte ratio
NT-proBNP: N-terminal pro-B-type natriuretic peptide
PAD4: Peptidylarginine deiminase 4
PRR: Pattern recognition receptor
RA: Rheumatoid arthritis
RAGE: Receptor for advanced glycation end products
ROS: Reactive oxygen species
S100A8/A9: S100 calcium-binding protein A8/A9
SLE: Systemic lupus erythematosus
STEMI: ST-segment elevation myocardial infarction
T2DM: Type 2 diabetes mellitus
TAC: Transverse aortic constriction
TLR4/9: Toll-like receptor 4/9
TNF-*α*: Tumor necrosis factor-alpha.
